# Element of Therapeutics

**Published:** 1878-06

**Authors:** 


*The Elements of Therapeutics.* A Clinical Guide to the action of Medicines. By Dr. C. Binz, Professor of Pharmacology in the University of Bonn. Translated by Edward I. Sparks, M. A. M. B. Oxon. New York: Wm. Wood & Co., 1878. Buffalo: H. H. Otis.

The author divides the work into twelve chapters under the heads: Nervine Medicines, whose special purpose is to produce a sedative effect, Nervine medicines whose main action is stimulant, Ætherial Oils, Demulcent Medicines, Astringents, Bitter and Sweet Alkaline medicines, remedies promoting tissue growth, Diathetic and Antiseptic medicines, Antiseptics proper, Medicines which reduce fever, Evacuant remedies, Caustics and mechanical remedies. The remedies do not seem to arrange themselves as readily under this system as one could wish, and the enumeration of the various pharmaceutical preparations as found in Germany, England, and the United States, tends to confuse rather than instruct. The author hopes that the comparison between the British and American Pharmacopœias, which this works renders possible, may lead to a simplification of the enormous superfluity of preparations of the same drug, which exists in several instances in both. As a whole, many American works excell it for practical use.

